# Educational inequalities in major depressive disorder prevalence, timing and duration among adults over the life course: a microsimulation analysis based on the Lifelines Cohort Study

**DOI:** 10.1093/eurpub/ckae066

**Published:** 2024-04-13

**Authors:** Alexander Lepe, Liza A Hoveling, Michaël Boissonneault, Joop A A de Beer, Sijmen A Reijneveld, Marlou L A de Kroon, Aart C Liefbroer

**Affiliations:** Department of Health Sciences, Community and Occupational Medicine, University Medical Center Groningen, University of Groningen, Groningen, The Netherlands; Department of Epidemiology, University Medical Center Groningen, University of Groningen, Groningen, The Netherlands; Netherlands Interdisciplinary Demographic Institute (NIDI)-KNAW, University of Groningen, The Hague, The Netherlands; Netherlands Interdisciplinary Demographic Institute (NIDI)-KNAW, University of Groningen, The Hague, The Netherlands; Department of Health Sciences, Community and Occupational Medicine, University Medical Center Groningen, University of Groningen, Groningen, The Netherlands; Department of Health Sciences, Community and Occupational Medicine, University Medical Center Groningen, University of Groningen, Groningen, The Netherlands; Environment and Health, Department of Public Health and Primary Care, KU Leuven, Leuven, Belgium; Department of Epidemiology, University Medical Center Groningen, University of Groningen, Groningen, The Netherlands; Netherlands Interdisciplinary Demographic Institute (NIDI)-KNAW, University of Groningen, The Hague, The Netherlands; Department of Sociology, Vrije Universiteit Amsterdam, Amsterdam, The Netherlands

## Abstract

**Background:**

Educational inequalities in major depressive disorder (MDD) pose a major challenge. Tackling this issue requires evidence on the long-term impact of intervening on modifiable factors, for example lifestyle and psychosocial factors. For this reason, we aimed to simulate the development of educational inequalities in MDD across the life course, and to estimate the potential impact of intervening on modifiable factors.

**Methods:**

We used data from the prospective Dutch Lifelines Cohort Study to estimate the required input for a continuous-time microsimulation. The microsimulation allowed us to project the development of educational inequalities in MDD between ages 18 and 65, and to assess the potential benefit of intervening on quality of social contacts, health literacy and smoking.

**Results:**

On average, an additional 19.1% of individuals with low education will ever experience MDD between ages 18 and 65 compared with those with high education. Additionally, individuals with low education generally will develop MDD 0.9 years earlier and spend 1.2 years more with MDD, than individuals with high education. Improving the quality of social contacts in individuals with low education produced the largest effect; it would reduce the inequalities in the prevalence, onset and duration of MDD by an average of 18.4%, 18.3% and 28.6%, respectively.

**Conclusions:**

Intervening on modifiable factors, particularly quality of social contacts, in individuals with low education could help reduce the estimated educational inequalities in MDD over the life course.

## Introduction

Major depressive disorder (MDD) is a loss of interest and persistent depressed mood for most of the day over 2 weeks.^1^ An estimated 6.4% of Europeans are currently suffering from depression.^2^ Within The Netherlands, nearly 20% of adults aged 18–64 years will ever experience depression, and the cost of depression care in 2015 was estimated to be 1.63 billion euros.^3^ Importantly, depression affects individuals with low education more than those with high education.^4^ Reducing these educational inequalities in MDD is an important public health issue.

Modifiable factors, which include psychosocial and lifestyle factors, may partially account for these educational inequalities,^5^ but there is a lack of evidence on the long-term impact of intervening on these factors. Generally, various studies have shown that individuals with low education have worse profiles for modifiable factors,^6–11^ and these modifiable factors are then associated with worse outcomes.^9^^,^^12–14^ Specifically, low education has been shown to result in less favourable quality of social contacts, health literacy and smoking patterns.^6^^,^^9^^,^^15^ Social contacts have been shown to reduce depression, and this may be explained by the fact that interpersonal relationships can help individuals cope with stress.^16^ Individuals with low health literacy have difficulties in preventing and managing chronic diseases,^17^ which may explain its influence on depression.^9^ The effects of smoking on depression may be partially due to nicotine desensitizing nicotinic acetylcholine receptors, which play a role in regulating mood disorders.^15^ Intervening upon these factors may help reduce educational inequalities in MDD, but evidence is needed on the impact of intervening upon these factors over the adult life course.

This study aims to simulate the educational inequalities in MDD over the adult life course, and to estimate the potential impact of intervening on modifiable factors. We accomplish this using microsimulation models, which allows the estimation of population-level outcomes from individual behaviours. Simulation parameters are based on empirical data from a large prospective population-based cohort. We estimate the life course prevalence, mean age at onset and mean duration using the simulation output. This analysis can also be used to assess how changes in modifiable factors affect these parameters, which can help inform future policy.

## Methods

### Study design and data source

We used a microsimulation model to estimate the educational inequalities in MDD over the adult life course, and to estimate the potential impact of intervening on modifiable factors. We used empirical data from the Lifelines Cohort Study to estimate the input parameters for our microsimulation model.^18^ Lifelines is a multidisciplinary prospective population-based cohort study examining in a unique three-generation design the health and health-related behaviours of 167 729 persons living in the North of The Netherlands. Lifelines employs a broad range of investigative procedures in assessing the biomedical, sociodemographic, behavioural, physical and psychological factors which contribute to the health and disease of the general population, with a special focus on multimorbidity and complex genetics. Details about the recruitment and the data collection are described elsewhere.^18^ Prior to joining the cohort, each participant provided informed consent. The Lifelines Cohort Study follows the conventions set forth in the Declaration of Helsinki. The University Medical Center Groningen’s Medical Ethics Committee has also approved of the Lifelines Cohort Study (approval number: 2007/152).

This study used data from 152 728 individuals aged 18 years and older. In total, 78 749 participants were excluded for one of the following reasons: age ≥66 years, missing MDD data, >30% of the variables were missing or lost to follow-up. The final sample consisted of 73 979 individuals, which were on average 43.4 years old (SD 11.0), and participated in the Lifelines cohort between 2007 and 2017. The sample included in the analysis tended to be slightly younger, more female, higher educated, and had more favourable modifiable factors than those excluded from the analysis (Supplementary table S1). However, these differences were generally small. The participation-to-prevalence ratio (PPR), which is the ratio between the proportion of a subgroup of participants to that among the total population,^19^ was 0.94 for individuals with junior general secondary (low) education and 1.07 for individuals with university (high) education (Supplementary table S1); a PPR between 0.8 and 1.2 represents adequate representation. Similarly, the PPR for females and the modifiable factors generally indicated adequate representation (ranging from 0.93 to 1.03). The difference in age was also small; individuals included in our sample were on average 2.4 years (Cohen’s *d* 0.18) younger than those excluded from our sample.

### Measures and procedures

Below, the operationalization of the dependent variable and the main independent variable is described. The way all items were measured in Lifelines and the operationalization of the modifiable factors is presented in Supplementary tables S2 and S3.

#### Education

Individuals reported their highest level of competed education, which was recoded into years of education using the number of years it would take to attain each educational level by the fastest route possible.^20^ The microsimulation compares two specific educational levels, junior general secondary education (10 years) and university education (16 years), which are referred to as low and high education, respectively.

#### Major depressive disorder

MDD was assessed with the Mini International Neuropsychiatric Interview (MINI). The MINI is a validated tool, which evaluates the presence of an episode of MDD in the past 2 weeks according to DSM-IV criteria.^1^ During the baseline assessment trained interviewers administered the MINI, and during the second assessment participants filled in a digital questionnaire.

### Modelling approach

Microsimulation was used to create a synthetic cohort with individual life courses that transition into and out of MDD. Transition rates are based on MDD incidence and remittance rates derived from our empirical data.^18^ The individual life courses are then characterized by the sequence of states and amount of time spent in each state over time. In this study, we first constructed life courses stratified by sex and education, which were used to estimate a set of population parameters. Due to educational differences across various modifiable risk factors, we then conduct a counterfactual analysis. This analysis allows us to consider the potential impact of intervening on these modifiable factors on educational inequalities in MDD.

#### Simulating educational inequalities in MDD across the life course

We developed a continuous-time microsimulation using the MicSim function from the MicSim package available in R (v4.0.2).^21^ This model simulates transitions into and out of MDD for individual life courses following continuous-time Markov chains. We simulated 500 000 unique life courses from the age of 18 until 65 years; mortality was not accounted for in the model. These life courses were evenly divided between males and females with low and high education. This sample was sufficient to remove the uncertainty associated with the model’s Monte Carlo component (Supplementary figures S1–S3).

To construct our simulation, we estimated three sets of rates with the Lifelines data. We estimated the prevalence of MDD at age 18 and both MDD incidence and remittance rates per 5-year age group between the age of 18–65 years; these rates were estimated using multivariable logistic regression models. Supplementary Appendix A details the procedure used to account for missing data and estimate these rates. Using the simulated life courses we then estimate the life course prevalence, mean age of onset and mean duration with MDD, separately by sex and educational level. *Life course prevalence* refers to the proportion of individuals who *ever* had MDD. *Mean age of onset* was defined as the average age at which individuals first develop MDD; only individuals who did not have MDD at the start of the simulation and experienced MDD at least once during the were included when calculating the mean age of onset. *Mean duration of MDD* was defined as the average number of years spent with MDD among individuals who experienced MDD.

#### Assessing the potential impact of intervening on modifiable factors

To assess the potential impact of intervening on modifiable factors we estimated counterfactual transition rates for the individuals with low education. This allows us to consider what the educational inequalities in MDD would look like if the distribution of the most important modifiable factors in individuals with low education mirrored that of in individuals with high education. The modifiable variables included in this analysis were occupational and leisure time moderate-to-vigorous physical activity, smoking, alcohol intake, diet quality, sleep duration, network size, quality of social contacts, partner status and health literacy. We identified the most important factors estimating the mediating percentages of these factors (see Supplementary Appendix B for details).

The most important modifiable factors were then included in the logistic regression models used to estimate the counterfactual transition rates. We estimated new transition rates for individuals with low education by adjusted their distribution of the modifiable factors to be the same as for individuals with high education. These counterfactual rates were then fed into the microsimulation where we followed the same procedure used to estimate educational inequalities in MDD over the life course. We estimated one counterfactual simulation per modifiable factor (3 in total). An additional simulation in which all modifiable factors were changed at once (joint effect) was also estimated.

#### Sensitivity analyses

In sensitivity analyses, we assessed the role of having a history of depression on the development of depression over the life course. To do so we estimated incidence and remittance rates using a subsample from Lifelines who reported a history of depression before the baseline assessment (Supplementary figure S5). These transition rates were used in the simulation after an individual remitted MDD once.

## Results

### Educational inequalities in MDD over the life course

**Figure 1:**
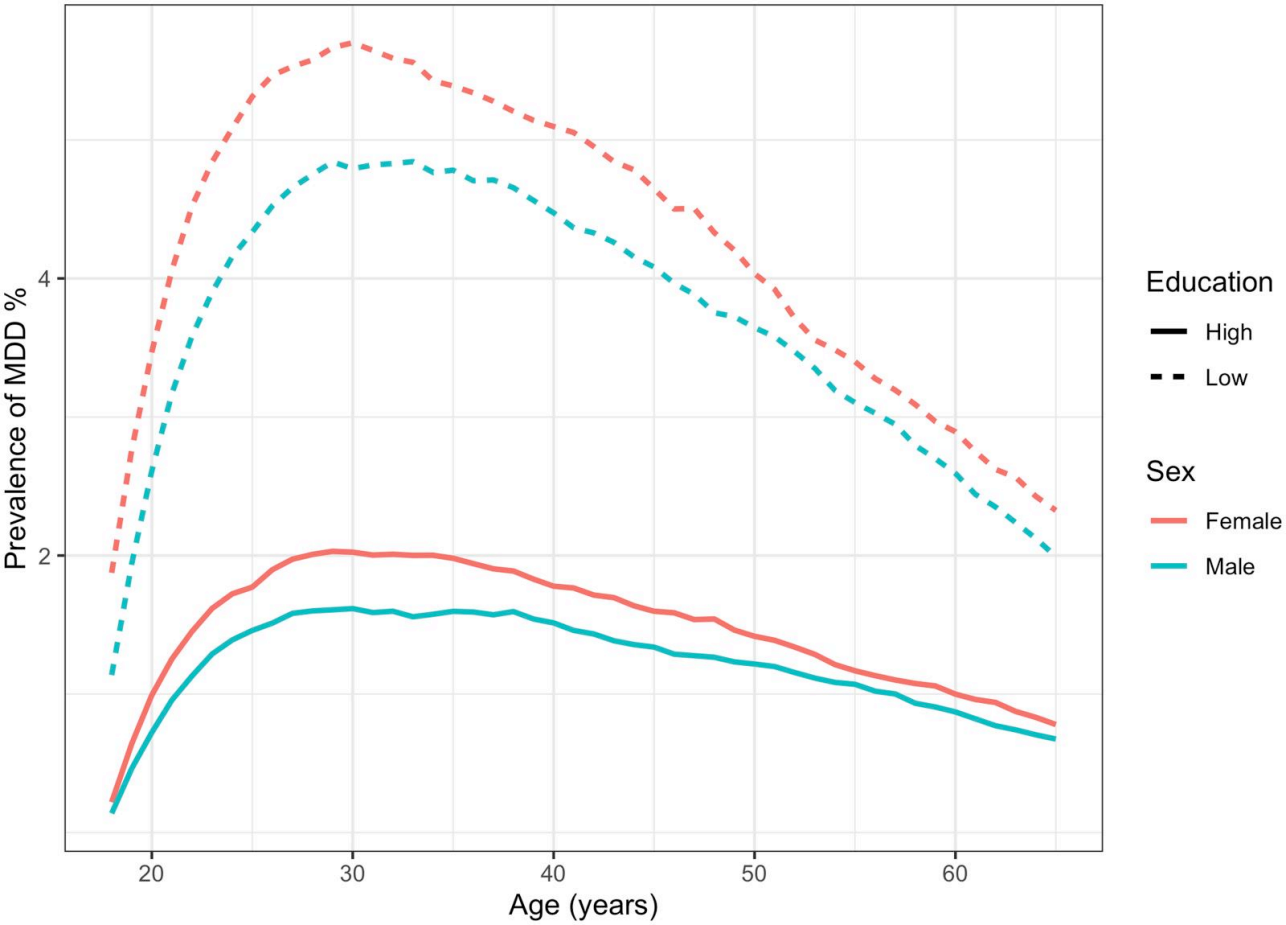
Age-specific prevalence of MDD stratified by sex and education. Model parameters estimated using data from adults in the Lifelines Cohort Study collected between 2007 and 2017.

Based on our microsimulation we found that individuals with low education are substantially more likely to ever experience MDD, experience MDD slightly earlier in life, and spend longer periods with MDD than individuals with high education (table 1); the model parameters were estimated using data from the adults participating in the Lifelines Cohort Study (data was collected between 2007 and 2017). Individuals with low education are more than twice as likely as those with high education to ever experience MDD between the ages of 18 and 65, 32.0% vs. 12.9%, respectively. Females with low education have a 20.8 percentage points higher life course prevalence of MDD than females with high education. In males, this difference is 17.3 percentage points. The life course prevalence of developing MDD is much higher than its age-specific prevalence, but the relative differences are larger for the age-specific prevalence (figure 1). At any point in time, less than six percent of adults experienced MDD, and on average individuals with low education are about 3 times more likely to have MDD than individuals with high education. Using the life course prevalence, this difference becomes 2.5 times.

**Table 1:** Educational inequalities in the development of MDD between ages 18 and 65 stratified by sex. Model parameters estimated using data from adults in the Lifelines Cohort Study collected between 2007 and 2017

Sex	Education	Life course prevalence (%)	Mean age of onset (years)	Mean duration (years)
Females	Low	35.3	35.5	5.8
	High	14.5	36.3	4.9
Males	Low	28.6	35.7	6.6
	High	11.3	36.6	5.1

On average, MDD onset occurs in the mid-30s (table 1), which is older than the peak in age-specific prevalence (figure 1). Females and males with low education on average develop MDD 0.8 and 0.9 years earlier and spend an additional 0.9 and 1.5 years with MDD compared with their highly educated counterparts, respectively. The sensitivity analysis results were generally similar to those from the primary analysis (Supplementary table S4).

### The potential impact of intervening on modifiable factors

**Figure 2:**
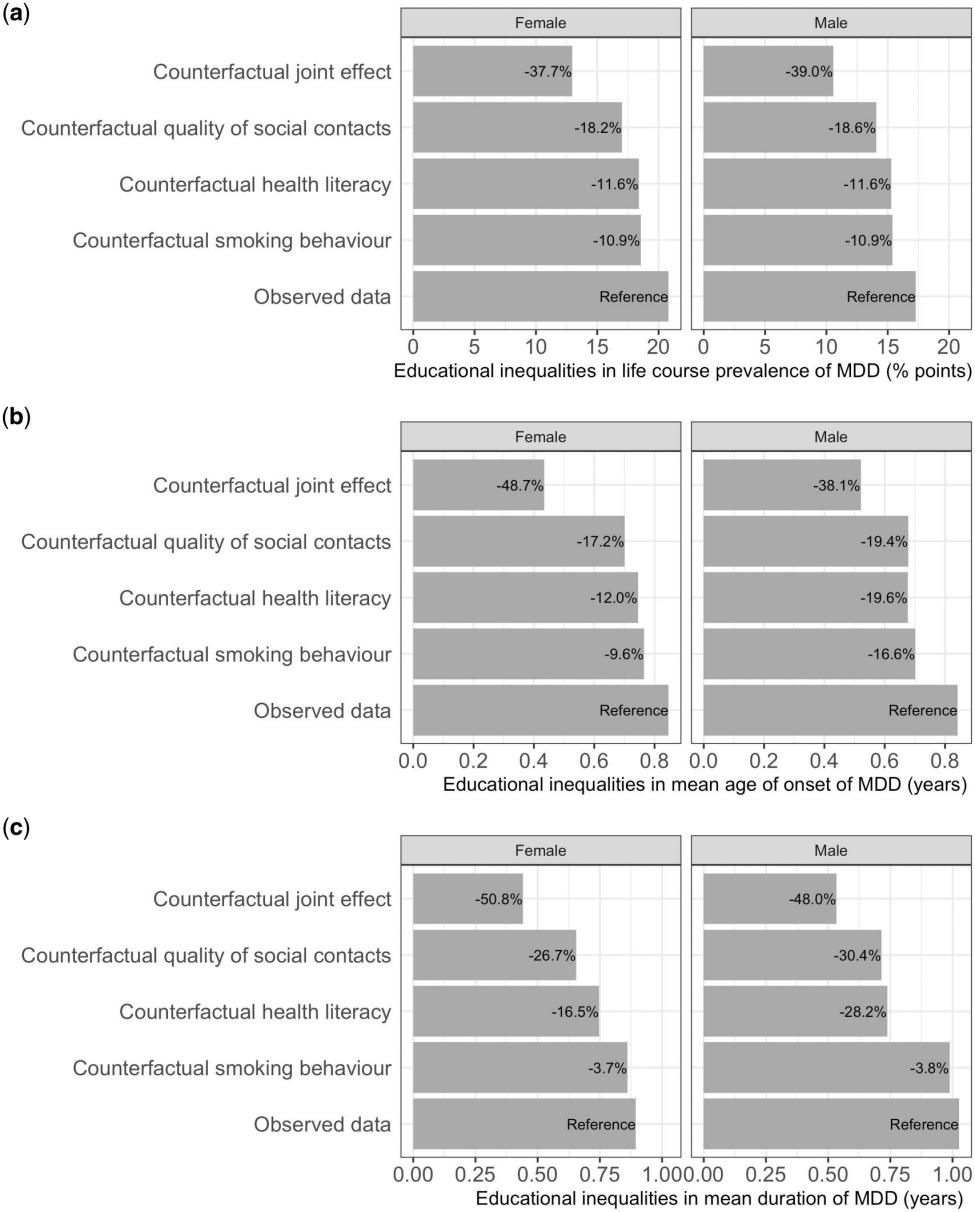
Potential impact of modifiable factors on educational inequalities in (a) life course prevalence, (b) mean age of onset of and (c) mean duration of MDD between ages 18 and 65. Model parameters estimated using data from adults in the Lifelines Cohort Study collected between 2007 and 2017. The bars show (a) the additional proportion of individuals with low education who ever experience MDD compared with individuals with high education, (b) the difference in the mean age of onset between individuals with low and high education and (c) the additional number of years spent with MDD for individuals with low education compared with individuals with high education under different counterfactual conditions. Larger values for the difference in mean age of onset indicate earlier age of onset among individuals with low education. The percentages shown on the bars represent the amount the educational inequalities would diminish under the given counterfactual.

Quality of social contacts, health literacy and smoking behaviour were identified as the most important modifiable factors (Supplementary tables S5–S10). The counterfactual simulations demonstrate that changes in these modifiable factors reduce the educational inequalities in life course prevalence, mean age of onset and mean duration of MDD (figure 2 and Supplementary table S11). However, changes in the mean age of onset and mean duration were not very large.

Among the independent modifiable factors, quality of social contacts had the largest potential impact. If quality of social contacts in females with low education were changed to reflect the patterns seen in females with high education, the life course prevalence of MDD would decrease from 35.3% to 31.5%. This decrease of 3.8% points represents an 18.2% reduction in the educational inequalities among females (table 1 and figure 2a). Similarly, for males, there is an 18.6% reduction in the educational inequalities. Changes to quality of social contacts would increase the mean age of onset of MDD in females with low education from 35.5–35.6 years, which is a 17.2% reduction in the educational inequalities (table 1 and figure 2b). In males, this is a 19.4% decrease. Similarly, changes to quality of social contacts would decrease the mean duration of MDD in females with low education from 5.8–5.5 years, which is a 26.7% reduction in the educational inequalities (table 1 and figure 2c). For males, there is a 30.4% reduction. Similarly, changes to health literacy and smoking behaviours also reduced educational inequalities in MDD, but to a lesser extent than changes in quality of social contacts (figure 2 and Supplementary table S11).

As expected, the educational differences in MDD would be smallest when all modifiable factors in individuals with low education were changed to reflect the patterns seen in individuals with high education. If all the modifiable factors were changed, the educational inequalities in life course prevalence, mean age of onset and mean duration of MDD would be reduced by 37.7%, 48.7% and 50.8% in females and 39.0%, 38.1% and 48.0% in males, respectively. The results from the sensitivity analysis (Supplementary figure S6 and table S12) were largely similar to those of the primary analysis.

## Discussion

We simulated educational inequalities in the development of MDD across the life course and assessed the influence of modifiable factors. We found that individuals with low education were substantially more likely to ever experience MDD, experienced MDD slightly earlier in life, and spent slightly longer periods with MDD than individuals with high education. Importantly, changes in the modifiable factors, particularly quality of social contacts, resulted in a significant reduction in educational inequalities in MDD. These estimates are based on a microsimulation model that was constructed using data from the adults participating in the Lifelines Cohort Study (data collection occurred between 2007 and 2017).

Our findings on the population-level parameters of MDD are generally in line with previous research. Dutch estimates, which used data collected between 2007 and 2009, have found that almost 20% of adults aged 18–64 will ever experience depression and the annual prevalence is about 5%,^3^ which coincides with our estimates. Recent age-specific estimates of the prevalence of depression, which are based on data collected from 2019 to 2022, show that the prevalence is largest among those aged 18–24.^22^ The age-specific prevalence remains nearly unchanged in individuals aged 25–34, and then similar to our results begins to decline as individuals age. The earlier peak in the prevalence of depression may be due to the increasing rates of depression, which may have affected the younger cohorts more.^23^ Our findings add to the body of evidence showing an educational gradient in the prevalence of MDD.^4^ The educational gradient in the prevalence of MDD over the life course was slightly smaller than the educational gradient at each age. This difference is partly because educational differences in the prevalence of MDD over the life course do not only depend on differences in the incidence but also differences in remittance. Our findings regarding the mean onset and duration of MDD coincide with evidence that individuals in The Netherlands with lower education have shorter healthy life expectancies compared with individuals with high education.^24^ Last, our findings on the mean duration of MDD are also in line with previous research which found that low socioeconomic position decreased the odds of remitting MDD.^25^

Approximately 40% of the educational inequalities in MDD were related to differences in quality of social contacts, health literacy and smoking. Changes in quality of social contacts had the largest impact in reducing educational inequalities in the prevalence, timing and duration of MDD. This is in line with a study that investigated the role of various mediators in the incidence of (symptoms of) MDD and found that psychosocial factors were more important than physical health or behavioural factors.^12^

This study’s strengths lie in the use of microsimulation and in the quality of its data. Microsimulation provides insights for policy making which traditional epidemiological methods may not offer. Furthermore, the robustness of our findings was supported by our sensitivity analysis, which yielded the same pattern of results as our primary analysis. We also make use of high-quality data from Lifelines, which is a population-based prospective cohort that follows standardized protocols for data collection, to estimate our simulation parameters.^26^

A limitation of this study stems from the differences between those included and excluded from the analysis. The individuals excluded from the sample used to estimate the rates used to build the simulation tended to be slightly older, contained a smaller proportion of females, were less educated, and had less favourable modifiable factors. Therefore, we may have missed some of the worst levels in both educational level and the modifiable factors, which may lead to an underestimation of the educational inequalities in MDD. However, these differences were small and overall we had adequate representation of the different groups in our analysis. Therefore, we do not expect this to have had a large impact on our results. We could not account for all of the important mechanisms that link education to MDD, such as occupational factors.^27^ Nonetheless, we were still able to identify some potentially important modifiable targets. Last, changing the way the MINI questionnaire was administered may have introduced some measurement error. If social desirability bias were stronger for the interview than the self-administered questionnaire, we may have underestimated the number of individuals with depression during the baseline assessment. This may have then lead to an overestimation of our incidence rates, which would inflate our estimates for the life course prevalence of MDD. However, the MINI has been validated when administered as an interview,^1^ and our estimates for the life course prevalence of MDD are similar to other Dutch estimates from a similar time period.^3^ Therefore, we do not expect this to have had a great influence on our results.

Additionally, our microsimulation models are based on several assumptions that should be scrutinized. First, we assumed that the transition rates we estimated will not change in the future. Second, we only compared two common educational levels in The Netherlands. Including additional educational levels would result in more refined insights into the effect of smaller educational differences. Last, mortality was not included in our models. MDD has been linked with higher mortality.^28^ Therefore, if we included mortality, the educational inequalities in the duration of MDD would likely be smaller. However, this decrease would be due to an increase in the life years lost among individuals with low education and not due to spending more years of life without MDD. The omission of mortality is mitigated by the fact that we limited our analyses to individuals aged 18–65, which is an age most individuals in The Netherlands reach.^24^

The results of this study can have important implications for various stakeholders. We found large educational inequalities in MDD using data from The Netherlands, which has a rather strong welfare state and highly subsidized health care system. These inequalities are potentially larger in countries that lack such well-developed social safety nets. Additionally, recent studies have also shown that the prevalence of depression has increased in recent years,^23^ and it is likely that this increase disproportionally affects individuals with low education.^4^ Therefore, our estimates may be underestimating both the overall prevalence of MDD and the size of the educational inequalities. Overall, this stresses the need to address educational inequalities in MDD. One strategy would be to improve educational attainment in vulnerable groups. Improving educational attainment would likely decrease the burden of MDD, but it should be noted that increasing educational attainment past tertiary education may not provide any additional benefit.^29^ Another strategy would be to offer tailor-made interventions and treatments for individuals with low education. This strategy has the benefit of prioritizing those who are most in need of assistance. For instance, our results suggest that interventions improving the quality of social contact could be particularly beneficial in reducing the burden of MDD. One approach to improving quality of social contacts is through school-based interventions. For example, a recent review found that school-based interpersonal psychotherapy—adolescent skills training may help young people at risk of depression.^30^ Incorporating health literacy into schooling could improve health literacy in the general population, and it could empower individuals to make better choices.^31^ These school-based interventions early in life have the potential to improve various aspects of health and well-being throughout the life course.

## Conclusion

There are large educational inequalities in MDD over the life course. Compared with individuals with high education, individuals with low education are substantially more likely to ever experience MDD, experience MDD earlier in life, and spend longer periods with MDD. These inequalities may be reduced by improving modifiable factors, particularly quality of social contacts. Additional research is needed to assess what other factors contribute to these educational inequalities.

## Supplementary Material

ckae066_Supplementary_Data

## Data Availability

The generated dataset is not publicly available as it is created and used under license from the Lifelines Cohort Study. Data from the Lifelines Cohort Study is available on request (https://www.lifelines.nl/researcher). However, the transition rates which were estimated using the Lifelines dataset and serve as the input for the microsimulation models are available online; the code for the estimation of the prevalence rates, transition rates and microsimulation models is also available online (https://github.com/a-lepe/TRANSSES_mdd_simulation).

## References

[1] Sheehan DV , Lecrubier Y, Sheehan KH, et al The Mini-International Neuropsychiatric Interview (M.I.N.I.): the development and validation of a structured diagnostic psychiatric interview for DSM-IV and ICD-10. J Clin Psychiatry 1998;59 Suppl 20:22–33. 9881538

[2] Arias-De La Torre J , Vilagut G, Ronaldson A, et al Prevalence and variability of current depressive disorder in 27 European countries: a population-based study. Lancet Public Health 2021;6:e729–38. 33961802 10.1016/S2468-2667(21)00047-5 PMC8460452

[3] Nuijen J , van Bon-Marten M, de Graaf R, et al Zicht op Depressie: De Aandoening, Preventie en Zorg. Themarapportage Van de Staat Van Volksgezondheid en Zorg [A Clear View of Depression: The Disorder, Prevention and Care. Thematic Report of the State of Public Health and Care]. Bilthoven: National Institute for Public Health and the Environment (RIVM), 2018.

[4] Lorant V , Deliege D, Eaton W, et al Socioeconomic inequalities in depression: a meta-analysis. Am J Epidemiol 2003;157:98–112. 12522017 10.1093/aje/kwf182

[5] Hoveling LA , Liefbroer AC, Schweren LJS, et al Socioeconomic differences in major depressive disorder onset among adults are partially explained by lifestyle factors: a longitudinal analysis of the Lifelines Cohort Study. J Affect Disord 2022;314:309–17. 35850289 10.1016/j.jad.2022.06.018

[6] Ajrouch KJ , Blandon AY, Antonucci TC. Social networks among men and women: the effects of age and socioeconomic status. J Gerontol B Psychol Sci Soc Sci 2005;60:S311–7. 16260713 10.1093/geronb/60.6.s311

[7] Coenen P , Huysmans MA, Holtermann A, et al Can socioeconomic health differences be explained by physical activity at work and during leisure time? Rationale and protocol of the active worker individual participant meta-analysis. BMJ Open 2018;8:e023379. 10.1136/bmjopen-2018-023379 PMC6224722 30373782

[8] Newton S , Braithwaite D, Akinyemiju TF. Socio-economic status over the life course and obesity: systematic review and meta-analysis. PLoS One 2017;12:e0177151. 28510579 10.1371/journal.pone.0177151 PMC5433719

[9] Stormacq C , Van den Broucke S, Wosinski J. Does health literacy mediate the relationship between socioeconomic status and health disparities? Integrative review. Health Promot Int 2019;34:e1–17. 30107564 10.1093/heapro/day062

[10] Stringhini S , Haba-Rubio J, Marques-Vidal P, et al Association of socioeconomic status with sleep disturbances in the Swiss population-based CoLaus study. Sleep Med 2015;16:469–76. 25777484 10.1016/j.sleep.2014.12.014

[11] Stringhini S , Sabia S, Shipley M, et al Association of socioeconomic position with health behaviors and mortality. JAMA 2010;303:1159–66. 20332401 10.1001/jama.2010.297 PMC2918905

[12] Koster A , Bosma H, Kempen GI, et al Socioeconomic differences in incident depression in older adults: the role of psychosocial factors, physical health status, and behavioral factors. J Psychosom Res 2006;61:619–27. 17084139 10.1016/j.jpsychores.2006.05.009

[13] Sarris J , O’Neil A, Coulson CE, et al Lifestyle medicine for depression. BMC Psychiatry 2014;14:107. 24721040 10.1186/1471-244X-14-107 PMC3998225

[14] Schuch FB , Vancampfort D, Firth J, et al Physical activity and incident depression: a meta-analysis of prospective cohort studies. Am J Psychiatry 2018;175:631–48. 29690792 10.1176/appi.ajp.2018.17111194

[15] Treur JL , Munafò MR, Logtenberg E, et al Using Mendelian randomization analysis to better understand the relationship between mental health and substance use: a systematic review. Psychol Med 2021;51:1593–624. 34030749 10.1017/S003329172100180X PMC8327626

[16] Kuczynski AM , Halvorson MA, Slater LR, et al The effect of social interaction quantity and quality on depressed mood and loneliness: a daily diary study. Journal of Soc Pers Relat 2022;39:734–56.

[17] Chew LD , Bradley KA, Boyko EJ. Brief questions to identify patients with inadequate health literacy. Fam Med 2004;36:588–94. 15343421

[18] Scholtens S , Smidt N, Swertz MA, et al Cohort Profile: lifeLines, a three-generation cohort study and biobank. Int J Epidemiol 2015;44:1172–80. 25502107 10.1093/ije/dyu229

[19] Poon R , Khanijow K, Umarjee S, et al Participation of women and sex analyses in late-phase clinical trials of new molecular entity drugs and biologics approved by the FDA in 2007–2009. J Womens Health (Larchmt) 2013;22:604–16. 23768021 10.1089/jwh.2012.3753 PMC3704049

[20] De Graaf ND , De Graaf PM, Kraaykamp G. Parental cultural capital and educational attainment in The Netherlands: a refinement of the cultural capital perspective. Sociol Educ 2000;73:92–111.

[21] Zinn S. The MicSim package of R: an entry-level toolkit for continuous-time microsimulation. IJM 2013;7:3–32.

[22] ten Have M , Tuithof M, van Dorsselaer S, et al NEMESIS, de psychische gezondheid van de Nederlandse bevolking [NEMESIS, the mental health of the Dutch population]. Trimbos-instituut. Available at: https://cijfers.trimbos.nl/nemesis/verdiepende-informatie-psychische-aandoeningen/depressieve-stoornis/ Updated 08 November 2023 (7 February 2024, last date accessed).

[23] ten Have M , Tuithof M, van Dorsselaer S, et al Prevalence and trends of common mental disorders from 2007-2009 to 2019-2022: results from the Netherlands Mental Health Survey and Incidence Studies (NEMESIS), including comparison of prevalence rates before vs. during the COVID-19 pandemic. World Psychiatry 2023;22:275–85. 37159351 10.1002/wps.21087 PMC10168151

[24] Statistics Netherlands. Life Expectancy; Sex; Age. Available at: https://www.cbs.nl/nl-nl/cijfers/detail/84842NED#shortTableDescription Published 2021. (10 December 2021, last date accessed).

[25] Kelly KM , Mezuk B. Predictors of remission from generalized anxiety disorder and major depressive disorder. J Affect Disord 2017;208:467–74. 27863710 10.1016/j.jad.2016.10.042 PMC5515235

[26] Klijs B , Scholtens S, Mandemakers JJ, et al Representativeness of the LifeLines Cohort Study. PLoS One 2015;10:e0137203. 26333164 10.1371/journal.pone.0137203 PMC4557968

[27] Niedhammer I , Sultan-Taïeb H, Parent-Thirion A, et al Update of the fractions of cardiovascular diseases and mental disorders attributable to psychosocial work factors in Europe. Int Arch Occup Environ Health 2022;95:233–47. 34181059 10.1007/s00420-021-01737-4 PMC8237556

[28] Cuijpers P , Smit F. Excess mortality in depression: a meta-analysis of community studies. J Affect Disord 2002;72:227–36. 12450639 10.1016/s0165-0327(01)00413-x

[29] Chlapecka A , Kagstrom A, Cermakova P. Educational attainment inequalities in depressive symptoms in more than 100,000 individuals in Europe. Eur Psychiatry 2020;63:e97. 33190666 10.1192/j.eurpsy.2020.100 PMC7737177

[30] Filia K , Eastwood O, Herniman S, et al Facilitating improvements in young people's social relationships to prevent or treat depression: A review of empirically supported interventions. Transl Psychiatry 2021;11:305. 34021113 10.1038/s41398-021-01406-7 PMC8139977

[31] Okan O , Paakkari L, Dadaczynski K. Health Literacy in Schools: State of the Art SHE Factsheet No. 6. Denmark: Schools for Health in Europe, 2020.

