# Supplementary material for: Educational inequalities in major depressive disorder prevalence, timing and duration among adults over the life course: a microsimulation analysis based on the Lifelines Cohort Study
Source: Eur J Public Health. 2024 Apr 13;34(4):723–9. doi: 10.1093/eurpub/ckae066 (PMC11293816; doi:10.1093/eurpub/ckae066)
Supplement: ckae066_Supplementary_Data [file ckae066_supplementary_data.pdf]

SUPPLEMENTARY MATERIAL

1. **Appendix A.** Description of the process used to estimate and parameterize the rates for the microsimulations

2. **Appendix B.** Description of the process used to estimate the mediating percentages used to derive the most important modifiable factors

3. **Supplementary Tables**

Table S1. Descriptive characteristics of the study population with and without MDD at baseline (n=73,979).

Table S2. Measurement in the Lifelines Cohort Study of the variables used in the analyses.

Table S3. Operationalisation of the modifiable variables included in the mediation analyses.

Table S4. Educational inequalities in the development of MDD over the life course stratified by sex. Sensitivity analysis using transition rates derived from a sample with a history of depression. Model parameters estimated using data from adults in the Lifelines Cohort Study collected between 2007 and 2017.

Table S5. Multivariable logistic regression analysis of the direct associations between education, lifestyle, social, and psychological factors and incident major depressive disorder.

Table S6. Multivariable mediation analysis of lifestyle, social, and psychological factors in the associations between education and incident major depressive disorder using the Karlson-Holm-Breen method.

Table S7. Multivariable logistic regression analysis of the direct associations between education, lifestyle, social, and psychological factors and remitted major depressive disorder.

Table S8. Multivariable mediation analysis of lifestyle, social, and psychological factors in the associations between education and remitted major depressive disorder using the Karlson-Holm-Breen method.

Table S9. Multivariable logistic regression analysis of the direct associations between education and the most important mediating factors and incident and remitted major depressive disorder.

Table S10. Multivariable mediation analysis of the most important mediating factors in the association between education and incident and remitted major depressive disorder using the Karlson-Holm-Breen method.

Table S11. Educational differences in the development of MDD over the life course stratified by sex, estimates based on the simulation. Model parameters estimated using data from adults in the Lifelines Cohort Study collected between 2007 and 2017.

Table S12. Educational differences in the development of MDD over the life course stratified by sex. Sensitivity analysis using transition rates derived from a sample with a history of depression. Model parameters estimated using data from adults in the Lifelines Cohort Study collected between 2007 and 2017.

Table S13. Descriptive characteristics of the study population used in the sensitivity analyses with and without MDD at baseline (n=7,336).

4. Supplementary Figures

Fig S1. Monte Carlo variance associated with the estimates of the life course prevalence of MDD.

Fig S2. Monte Carlo variance associated with the estimates of the mean age of onset of MDD.

Fig S3. Monte Carlo variance associated with the estimates of the mean duration of MDD.

Fig S4. Flowchart of the population with and without MDD at baseline.

Fig S5. Flowchart of the population used for the sensitivity analysis.

Fig S6. Potential impact of modifiable factors on educational inequalities in a) life course prevalence, b) mean age of onset of, and c) mean duration of MDD between ages 18 and 65. Sensitivity analysis using transition rates derived from a sample with a history of depression. Model parameters estimated using data from adults in the Lifelines Cohort Study collected between 2007 and 2017.

Fig S7. Observed (points) and expected (lines) values of transition rates for males and females by education level. Model parameters estimated using data from adults in the Lifelines Cohort Study collected between 2007 and 2017.

1. Appendix A. Description of the process used to handle missing data and estimate the rates for the microsimulations

A description of the samples used for both the primary and sensitivity analyses including the amount of missing data can be found in Supplementary Tables 1 and 13, respectively. Missing values were imputed using the Multiple Imputation by Chained Equation method (10 imputed samples were drawn every 100 iterations).[32] The imputation model included the independent variables, baseline age, sex, the modifiable variables and the dependent variables.

This data was used to estimate the sex- and education- specific prevalence of MDD at the age of 18 years. This prevalence was calculated by estimating the probability of MDD at baseline in the group of participants ≤27 years for participants aged 18 years, per sex (male or female) and per education group (10 or 14 years) using logistic regression models. The model takes the form:

$$log\left(\frac{P(MDD)}{1-P(MDD)}\right) = a + b1 * Age + b2 * Sex + b3 * Education$$

This data was also used to estimate the age-, sex-, and education-specific incidence and remittance rates of MDD using 5-year age groups. The incidence and remittance rates were estimated using logistic regression models which included age, sex, education, quality of social contacts, health literacy, and smoking status as predictors of MDD status during the second assessment.

As the microsimulation models depend on continuous measures of age these transition rates were then parameterized to account for continuous age. The type of parametric model for each transition rate was chosen based on the goodness of fit of the model with the observed data. Goodness of fit was assessed by visual inspection of the graph of the model and observed data (Supplementary Figure 7). The best fitting models were quadratic curves for remittance rates (mean absolute error: females with high education 0.0002; females with low education 0.0006; males with high education 0.0002; males with low education 0.0005), and linear functions for the incidence rates (mean absolute error: females with high education 0.0091; females with low education 0.0111; males with high education 0.0110; males with low education 0.0128).

2. Appendix B. Description of the process used to estimate the mediating percentages used to derive the most important modifiable factors

Population selection to estimate the mediating percentages

The current study used data from 152,728 participants aged 18 years and older. In total, 79,358 participants were excluded for one of the following reasons: aged over 65 years, MDD data was missing, missing data on more than 30% of the variables, or lost to follow-up. This resulted in a final sample of 73,370 participants, of which 72,033 did not have MDD at baseline and 1,337 had MDD at baseline.

Statistical analysis

Missing values were imputed using the Multiple Imputation by Chained Equation method (10 imputed samples were drawn every 100 iterations).[32] The imputation model included the independent variables, baseline age, sex, the modifiable variables (Supplementary Table 3), and the dependent variables. With the imputed datasets we then used a logistic regression model, which was adjusted for age, sex, and length of follow-up, to estimate the direct associations between education, the modifiable factors and incident/remitted MDD. The total, direct, and indirect associations between education and incident/remitted MDD via the modifiable factors and the mediating percentages of the modifiable factors were estimated using the Karlson-Holm-Breen (KHB) method.[33] The imputation of missing data and the mediation analyses were performed using StataMP 13 (64-bit).

3. Supplementary Tables

Table S1. Descriptive characteristics of the study population with and without MDD at baseline (n = 73,979).

**Characteristics**	**Lifelines population (n=152,728) [a]**	**Excluded population (n=78,749) [a]**	**Study population (n = 73,979) [a]**	**Population without MDD at baseline (n = 72,620) [a]**	**Population with MDD at baseline (n = 1,359) [a]**	**Participation to prevalence ratio (PPR) [b]**
Age (years), mean (SD)	44.6 (13.1)	45.8 (14.8)	43.4 (11.0)	43.4 (11.0)	43.0 (10.4)	-
Missing	0	0	0	0	0	
Sex (female)	58.5	57.2	59.8	59.6	70.5	1.02
Missing	0	0	0	0	0	
Education (years of education)						
Primary school not finished (5)	0.6	0.9	0.3	0.2	1.3	0.50
Primary education (6)	2.3	3.5	1.0	1.0	3.5	0.43
Lower or preparatory secondary vocational education (9)	13.3	15.5	11.0	10.9	18.8	0.82
Junior general secondary education (10)	13.6	14.3	12.9	12.8	17.7	0.94
Secondary vocational education or senior general secondary education (12)	38.5	36.1	41.1	41.1	39.4	1.06
Higher vocational education (15)	23.2	21.0	25.5	25.7	13.8	1.09
University education (16)	6.0	5.5	6.5	6.6	2.8	1.07
Missing	2.6	3.3	1.8	1.8	2.7	
**Modifiable factors**						
Smoking						
Never smoker	43.1	40.6	45.7	45.8	38.0	1.03
Past smoker	29.7	29.1	30.4	30.5	26.3	1.00
Current smoker	20.5	21.2	19.8	19.5	32.7	0.94
Missing	6.7	9.1	4.1	4.1	3.0	
Health literacy at T3 [c]						
Low	17.0	14.7	19.5	19.2	31.9	0.93
High	44.9	33.4	57.1	57.5	37.3	1.03

Missing	38.1	51.9	23.4	23.3	30.8	
Quality of social contacts [d]						
Low	28.1	27.0	29.3	28.7	60.8	0.99
Middle	35.0	32.3	38.0	38.3	20.4	1.03
High	29.4	28.3	30.6	30.9	15.3	0.98
Missing	7.5	12.4	2.2	2.2	3.5	

MDD: major depressive disorder; SD: standard deviation; [a] % are presented, unless indicated otherwise; [b] The PPR has been calculated for each level of the categorical variables by dividing the proportion of individuals included in our analysis by the proportion of individuals in the total sample. This calculation was done using the valid proportion of participants, i.e. excluding missing values; [c] Measured with the Brief Health Literacy Screening (BHLS) on a continuous scale (3-15) which was categorized in low health literacy 3-12 and high health literacy 13-15; [d] Measured with the Social Production Function Instrument (SPF-IL) on a continuous scale (0–27) which was categorized as ‘low’(< =14 points),‘middle’(15–17 points) and ‘high’(18–27 points).

Table S2. Measurement in the Lifelines Cohort Study of the variables used in the analyses.

**Variables**	**Measured in the Lifelines Cohort Study**
Education	Participants were asked, "what is the highest level of education you have completed?". They could then select from the following answer options: "No education", "Primary education", "Lower or preparatory secondary vocational education", "Junior general secondary education", "Secondary vocational education or work-based learning pathway", "Senior general secondary education, pre-university secondary education", "Higher vocational education" or "University education".
Major depressive disorder	The Mini International Neuropsychiatric Interview (MINI) was used to assess the presence of a current (in past two weeks) major depressive episode according to standard criteria in the Diagnostic Statistical Manual (DSM)-4.[1] The MINI was administered by trained interviewers at baseline, and in digital format (on-site) at the second assessment. The interview assesses two core symptoms: 1) consistently depressed or down; and 2) much less interested in most things. In addition, seven related symptoms were assessed: 3) significant weight loss (or poor appetite) or weight gain, 4) insomnia or hypersomnia, 5) psychomotor retardation, 6) fatigue or loss of energy, 7) feelings of worthlessness or excessive or inappropriate guilt, 8) diminished ability to think or concentrate, or indecisiveness, 9) recurrent thoughts of death, or suicidal ideation, plan, or attempt. For all of these symptoms, participants indicated their presence or absence. A major depressive episode is established when at least one main symptom and at least five symptoms in total are present.
Occupational moderate-to-vigorous physical activity (MVPA)	Measured using the Short QUestionnaire to Assess Health enhancing physical activity (SQUASH)[34] with questions about occupational, cycling, gardening and sports activities. Responses to the various items of this questionnaire were combined to estimate the total time spent on occupational MVPA.
Leisure time MVPA	Measured using the Short QUestionnaire to Assess Health enhancing physical activity (SQUASH)[34] with questions about occupational, cycling, gardening and sports activities. Responses to the various items of this questionnaire were combined to estimate the total time spent on leisure time MVPA.
Smoking	Participants were asked the following questions regarding their smoking status: "have you ever smoked for a full year?" and "do you smoke now, or have you smoked in the past month?". Responses to both questions were either "yes" or "no".
Alcohol	Measured using the Food Frequency Questionnaire (FFQ)[35] with two items. The first question was, "how often did you drink alcoholic drinks in the past month?". Respondents could select one of the following responses: "not this month", "1 day a month", "2-3 days a month", "1 day a week", "2-3 days a week", "4-5 days a week", or "6-7 days a week". Respondents who indicated they consumed alcohol in the past month were then asked, "how many glasses (i.e. alcoholic drinks) did you drink per day on average?". Respondents could respond any integer ranging from 1-11 or "12 or more".
Diet	The Lifelines Diet Score (LLDS) is based on the 2015 Dutch Dietary Guidelines[36] and measures the consumption of nine food groups (vegetables, fruit, whole grain products, legumes, nuts, fish, oils- and soft margarines, unsweetened dairy, coffee and tea) with positive and three food groups (red- and processed meat, butter- and hard margarines and sugar-sweetened beverages) with negative health effects.
Sleep	Participants were asked, "on average how many hours do you sleep per full day (24 hours)?". Participants gave a numeric response.
Network size	Participants were asked, "with on average how many different people do you have contact over a period of two weeks?". Participants gave a numeric response.
Quality of social contacts	The quality of social contacts was assessed using nine items from the Social Production Function Instrument for Level of Well-Being (SPF-IL) measuring 'affection', 'behavioural confirmation' and ´status´.[37] Participants were asked the following questions: "do people pay attention to you?", "do people help you if you have a problem?", "do you feel that people really love you?", "there are situations in which we deal with groups of people, for example at home, at work or during our leisure time. do others appreciate your role in the group?", "when you are at school, at work, with family, at an association or in church, do you feel like you

	belong?", "do others appreciate the things you do?", "do people think you do better than others?", "do people find you an influential person?", and "are you known for the things you have accomplished?". Answers to all items could be given on a four-point scale ranging from never (0) to always (3).
Partner status	Participants were asked, "do you have a partner?". Participants could give one of the following responses: "yes, I am married", "yes, I have a live-in partner or registered partnership", "yes, I am in a relationship but we are living apart/I am going out with someone", or "no, I am single / I have no partner".
Health literacy	Health literacy was measured with the Brief Health Literacy Screening (BHLS),[17] which consists of the following questions:<br>1. "How often do you have trouble understanding your medical situation because you have difficulty with the written information?"<br>2. "How sure are you of yourself when you fill out medical forms?"<br>3. "How often does someone help you with reading information materials from the hospital or another healthcare provider?"<br><br>Responses to these items were on a scale ranging from "never/not at all" (1) to "always/very" (5).

Table S3. Operationalisation of the modifiable variables included in the mediation analyses.

Variable	Operationalisation in this study
Occupational MVPA	We dichotomized the total time spent on occupational MVPA that was derived from the SQUASH.[34] This was dichotomized into those who did and did not complete at least 150 minutes of MVPA per week at work.[38]
Leisure time MVPA	We dichotomized the total time spent on leisure time MVPA that was derived from the SQUASH.[34] This was dichotomized into those who did and did not complete at least 150 minutes of MVPA per week during leisure time.[38]
Smoking	Responses from the two smoking questions were combined to create a single categorical variable with the following levels: never, former, or current smoker.
Alcohol intake	The two alcohol intake items from the FFQ were combined into one variable. Responses were categorized as “no alcohol intake”, “moderate alcohol intake”, or “excessive alcohol intake”. Moderate alcohol intake was defined as one glass of alcohol or less per day on average, without binge drinking (more than three glasses alcohol on one day for females and more than four glasses alcohol on one day for males). Excessive alcohol intake was defined as more than one glass of alcohol per day on average or binge drinking.[35,39]
Diet quality	Diet quality was based on the Lifelines Diet Score (LLDS) which was based on the 2015 Dutch Dietary Guidelines.[36,40] The LLDS is calculated as the sum of positive and negative food group quintile scores (range 0-48) and relative to the diet quality of the Lifelines population. Higher scores indicate a healthier diet. In the current study, there were no participants with a LLDS of 0 and therefore participants were divided into three groups, according to their LLDS, ‘poor’ diet quality (LLDS 1-16), ‘moderate’ diet quality (LLDS 17-32) or ‘high’ diet quality (LLDS 33-48).
Sleep duration	Sleep duration per full day was categorized as long (>9 hours), normal (7-9 hours) or short (<7 hours).
Network size	Network size was categorized as <5, 5-9, 10-14, ≥15 contacts
Quality of social contacts	Using the responses to the items from the SPF-IL, a sum score was calculated (range 0–27) with higher scores indicating higher social need fulfilment. Social need fulfilment was categorized as ‘low’(≤14 points),‘middle’(15–17 points) and ‘high’(≥18 points).
Partner status	Partner status at baseline was dichotomized to “yes” and “no”. To do so, all the responses that started with “yes” were combined into one level.
Health literacy	The participants’ responses to the first and third item of the BHLS were reversed and then summed with the responses to the second item. This led to a continuous scale (with scores ranging from 3 to 15) that was categorized as “low health literacy” (3-12) and “adequate health literacy” (13-15). The categorization for health literacy was based on the capturing of the non-linear relationship with education and resulted in a sufficient number of individuals in each category.

Table S4. Educational inequalities in the development of MDD over the life course stratified by sex. Sensitivity analysis using transition rates derived from a sample with a history of depression. Model parameters estimated using data from adults in the Lifelines Cohort Study collected between 2007 and 2017.

Sex	Education	Life course prevalence (%)	Mean age of onset (years)	Mean duration (years)
Females	Low	35.3	35.5	8.0
	High	14.5	36.3	5.8
Males	Low	28.6	35.7	8.6
	High	11.3	36.6	6.2

Table S5. Multivariable logistic regression analysis of the direct associations between education, lifestyle, social, and psychological factors and incident major depressive disorder.

	OR (95% CI)
**Path 1. Education → MDD**	0.87 (0.85, 0.89)*
**Path 2. Education → Lifestyle, social, and psychological factors**	
**Lifestyle factors**	
Occupational moderate-to-vigorous physical activity	
No (vs. Yes)	1.25 (1.23, 1.26)*
Leisure time moderate-to-vigorous physical activity	
No (vs. Yes)	0.95 (0.95, 0.96)*
Smoking (vs. Never)	
Former	0.93 (0.92, 0.94)*
Current	0.85 (0.84, 0.85)*
Alcohol intake (vs. Moderate)	
No	0.88 (0.87, 0.89)*
Excessive	0.96 (0.95, 0.96)*
Diet quality (vs. High)	
Moderate	0.87 (0.86, 0.88)*
Poor	0.74 (0.73, 0.76)*
Sleep duration (vs. 7-9)	
<7	0.99 (0.98, 1.00)*
>9	0.86 (0.83, 0.89)*
**Social factors**	
Network size (vs. 10-14)	
<5	0.89 (0.87, 0.90)*
5-9	0.97 (0.96, 0.98)*
≥15	1.01 (1.00, 1.02)
Quality of social contacts (vs. ≥18)	
≤14	0.87 (0.87, 0.88)*
15-17	0.97 (0.96, 0.98)*
Partner status	
No (vs. Yes)	0.96 (0.95, 0.97)*
**Psychological factors**	
Health literacy	
Low (vs. Adequate)	0.77 (0.76, 0.78)*
**Path 3. Lifestyle, social, and psychological factors → MDD**	
**Lifestyle factors**	
Occupational moderate-to-vigorous physical activity	
No (vs. Yes)	1.07 (0.95, 1.21)
Leisure time moderate-to-vigorous physical activity	
No (vs. Yes)	1.08 (0.98, 1.19)
Smoking (vs. Never)	
Former	1.15 (1.02, 1.31)*
Current	1.78 (1.59, 2.01)*
Alcohol intake (vs. Moderate)	

No	1.33 (1.17, 1.50)*
Excessive	0.98 (0.87, 1.10)
Diet quality (vs. High)	
Moderate	0.89 (0.74, 1.08)
Poor	1.09 (0.86, 1.37)
Sleep duration (vs. 7-9)	
<7	1.59 (1.39, 1.82)*
>9	1.91 (1.37, 2.66)*
**Social factors**	
Network size (vs. 10-14)	
<5	1.27 (1.08, 1.49)*
5-9	1.04 (0.91, 1.20)
≥15	0.88 (0.78, 0.99)*
Quality of social contacts (vs. ≥18)	
≤14	2.51 (2.21, 2.86)*
15-17	1.20 (1.05, 1.38)*
Partner status	
No (vs. Yes)	1.65 (1.47, 1.86)*
**Psychological factor**	
Health literacy	
Low (vs. Adequate)	1.66 (1.48, 1.86)*
**Path 4. Education → MDD controlled for lifestyle, social, and psychological factors**	0.93 (0.91, 0.95)*
Lifestyle factors	0.89 (0.87, 0.91)*
Social factors	0.89 (0.87, 0.91)*
Psychological factor	0.89 (0.87, 0.91)*

OR: odds ratio; CI: confidence interval; MDD: major depressive disorder; the analyses were controlled for age and sex at baseline and time between baseline and the second assessment. Reference categories for the lifestyle factors were occupational physical active, leisure time physical active, never smoker, moderate alcohol intake, high diet quality and 7-9 hours of sleep, reference categories for the social factors were a network of 10-14 persons, ≥18 points on the Social Production Function Instrument and having a partner, and reference categories for the psychological factor was 13-15 points on the Brief Health Literacy Screening; * P<0.05.

Table S6. Multivariable mediation analysis of lifestyle, social, and psychological factors in the associations between education and incident major depressive disorder using the Karlson-Holm-Breen method.

	OR (95% CI)
**Lifestyle factors**	
Total association	0.87 (0.85, 0.89)*
Direct association	0.89 (0.87, 0.91)*
Indirect association	0.98 (0.97, 0.99)*
Mediating effects (%)	
Total	14.1
Occupational moderate-to-vigorous physical activity	-1.5
Leisure time moderate-to-vigorous physical activity	0.6
Smoking	9.5
Alcohol intake	3.0
Diet quality	1.3
Sleep duration	1.2
**Social factors**	
Total association	0.87 (0.85, 0.89)*
Direct association	0.89 (0.87, 0.91)*
Indirect association	0.97 (0.97, 0.98)*
Total	19.5
Network size	2.8
Quality of social contacts	14.9
Partner status	1.8
**Psychological factors**	
Total association	0.87 (0.85, 0.89)*
Direct association	0.89 (0.87, 0.91)*
Indirect association	0.98 (0.97, 0.98)*
Total	16.9
Health literacy	16.9

OR: odds ratio; CI: confidence interval; MDD: major depressive disorder; the analyses were controlled for age and sex at baseline and time between baseline and the second assessment; * P<0.05

Table S7. Multivariable logistic regression analysis of the direct associations between education, lifestyle, social, and psychological factors and remitted major depressive disorder.

	OR (95% CI)
**Path 1. Education → MDD**	1.13 (1.07, 1.19)*
**Path 2. Education → Lifestyle, social, and psychological factors**	
**Lifestyle factors**	
Occupational moderate-to-vigorous physical activity	
No (vs. Yes)	1.14 (1.07, 1.22)*
Leisure time moderate-to-vigorous physical activity	
No (vs. Yes)	0.95 (0.91, 1.00)
Smoking (vs. Never)	
Former	0.94 (0.88, 0.99)
Current	0.85 (0.80, 0.90)*
Alcohol intake (vs. Moderate)	
No	0.86 (0.81, 0.91)*
Excessive	0.94 (0.88, 0.99)*
Diet quality (vs. High)	
Moderate	0.85 (0.77, 0.93)*
Poor	0.74 (0.65, 0.83)*
Sleep duration (vs. 7-9)	
<7	0.96 (0.91, 1.02)
>9	0.97 (0.88, 1.07)
**Social factors**	
Network size (vs. 10-14)	
<5	0.92 (0.86, 0.99)*
5-9	0.97 (0.91, 1.04)
≥15	0.99 (0.93, 1.05)
Quality of social contacts (vs. ≥18)	
≤14	0.91 (0.85, 0.97)*
15-17	1.02 (0.94, 1.10)
Partner status	
No (vs. Yes)	1.00 (0.95, 1.06)
**Psychological factors**	
Health literacy	
Low (vs. Adequate)	0.79 (0.74, 0.84)*
**Path 3. Lifestyle, social, and psychological factors → MDD**	
**Lifestyle factors**	
Occupational moderate-to-vigorous physical activity	
No (vs. Yes)	1.00 (0.69, 1.45)
Leisure time moderate-to-vigorous physical activity	
No (vs. Yes)	1.04 (0.79, 1.35)
Smoking (vs. Never)	
Former	1.47 (1.05, 2.07)*
Current	1.01 (0.75, 1.37)
Alcohol intake (vs. Moderate)	

No	0.63 (0.47, 0.86)*
Excessive	0.79 (0.57, 1.10)
Diet quality (vs. High)	
Moderate	0.76 (0.43, 1.35)
Poor	0.55 (0.28, 1.08)
Sleep duration (vs. 7-9)	
<7	0.84 (0.61, 1.14)
>9	0.45 (0.28, 0.72)*
**Social factors**	
Network size (vs. 10-14)	
<5	0.73 (0.50, 1.06)
5-9	0.76 (0.53, 1.08)
≥15	1.20 (0.85, 1.71)
Quality of social contacts (vs. ≥18)	
≤14	0.67 (0.46, 0.98)*
15-17	1.03 (0.65, 1.62)
Partner status	
No (vs. Yes)	0.75 (0.57, 1.00)*
**Psychological factors**	
Health literacy	
Low (vs. Adequate)	0.69 (0.51, 0.93)*
**Path 4. Education → MDD controlled for lifestyle, social, and psychological factors**	1.08 (1.02, 1.15)*
Lifestyle factors	1.11 (1.05, 1.17)*
Social factors	1.12 (1.06, 1.18)*
Psychological factors	1.11 (1.05, 1.17)*

OR: odds ratio; CI: confidence interval; MDD: major depressive disorder; the analyses were controlled for age and sex at baseline and time between baseline and the second assessment; reference categories for the lifestyle factors were occupational physical active, leisure time physical active, never smoker, moderate alcohol intake, high diet quality and 7-9 hours of sleep, reference categories for the social factors were a network of 10-14 persons, ≥18 points on the Social Production Function Instrument and having a partner, and reference categories for the psychological factor was 13-15 points on the Brief Health Literacy Screening; * P<0.05.

Table S8. Multivariable mediation analysis of lifestyle, social, and psychological factors in the associations between education and remitted major depressive disorder using the Karlson-Holm-Breen method.

	OR (95% CI)
**Lifestyle factors**	
Total association	1.13 (1.07, 1.20)*
Direct association	1.11 (1.05, 1.17)*
Indirect association	1.02 (1.00, 1.04)*
Mediating effects (%)	
Total	17.4
Occupational moderate-to-vigorous physical activity	0.0
Leisure time moderate-to-vigorous physical activity	-0.3
Smoking	0.0
Alcohol intake	9.0
Diet quality	7.0
Sleep duration	1.8
**Social factors**	
Total association	1.13 (1.07, 1.20)*
Direct association	1.12 (1.06, 1.18)*
Indirect association	1.01 (1.00, 1.03)*
Total	11.2
Network size	3.4
Quality of social contacts	7.8
Partner status	0.0
**Psychological factors**	
Total association	1.13 (1.07, 1.19)*
Direct association	1.11 (1.05, 1.17)*
Indirect association	1.02 (1.00, 1.04)*
Total	15.0
Health literacy	15.0

OR: odds ratio; CI: confidence interval; MDD: major depressive disorder; the analyses were controlled for age and sex at baseline and time between baseline and the second assessment; * P<0.05.

Table S9. Multivariable logistic regression analysis of the direct associations between education and the most important mediating factors and incident and remitted major depressive disorder.

	MDD incidence	MDD remission
	OR (95% CI)	OR (95% CI)
**Path 1. Education → Outcome**	0.87 (0.85, 0.89)*	1.13 (1.07, 1.19)*
**Path 2. Education → most important mediating factors**		
Smoking (vs. Never)		
Former	0.93 (0.92, 0.94)*	0.94 (0.88, 0.99)*
Current	0.85 (0.84, 0.85)*	0.85 (0.80, 0.90)*
Quality of social contacts (vs. ≥18)		
≤14	0.87 (0.87, 0.88)*	0.91 (0.85, 0.97)*
15-17	0.97 (0.96, 0.98)*	1.02 (0.94, 1.10)
Health literacy		
Low (vs. Adequate)	0.77 (0.76, 0.78)*	0.79 (0.74, 0.84)*
**Path 3. Most important mediating factors → Outcome**		
Smoking (vs. Never)		
Former	1.12 (0.99, 1.27)	1.50 (1.08, 2.09)*
Current	1.83 (1.63, 2.05)*	1.02 (0.76, 1.37)
Quality of social contacts (vs. ≥18)		
≤14	2.59 (2.28, 2.95)*	0.60 (0.41, 0.88)*
15-17	1.21 (1.06, 1.39)*	0.99 (0.63, 1.56)
Health literacy		
Low (vs. Adequate)	1.53 (1.36, 1.71)*	0.72 (0.53, 0.99)*
**Path 4. Education → Outcome controlled for most important mediating factors**	0.92 (0.90, 0.94)*	1.10 (1.04, 1.17)*

OR: odds ratio; CI: confidence interval; MDD: major depressive disorder; the analyses were controlled for age and sex at baseline and time between baseline and the second assessment; reference categories were never smoker, moderate alcohol intake, ≥18 points on the Social Production Function Instrument and 13-15 points on the Brief Health Literacy Screening; * P<0.05.

Table S10. Multivariable mediation analysis of the most important mediating factors in the association between education and incident and remitted major depressive disorder using the Karlson-Holm-Breen method.

	MDD incidence	MDD remission
	**OR (95% CI)**	**OR (95% CI)**
Total association	0.87 (0.85, 0.89)*	1.13 (1.07, 1.20)*
Direct association	0.92 (0.90, 0.94)*	1.10 (1.04, 1.17)*
Indirect association	0.95 (0.94, 0.95)*	1.03 (1.01, 1.05)*
Mediating effects (%)		
Total	39.6	23.2
Smoking	9.7	-0.2
Health literacy	15.7	9.4
Quality of social contacts	14.2	14.1

OR: odds ratio; CI: confidence interval; MDD: major depressive disorder; the analyses were controlled for age and sex at baseline and time between baseline and the second assessment; * P<0.05.

Table S11. Educational differences in the development of MDD over the life course stratified by sex, estimates based on the simulation. Model parameters estimated using data from adults in the Lifelines Cohort Study collected between 2007 and 2017.

Sex	Simulation*	Difference [a] in life course prevalence (% points)	Percentage [b] change in counterfactual	Difference [a] in mean age of onset (years)	Percentage [b] change in counterfactual	Difference [a] in mean duration (years)	Percentage [b] change in counterfactual
Females							
	Counterfactual joint effect	13.0	37.7	0.4	48.7	0.4	50.8
	Counterfactual quality of social contacts	17.0	18.2	0.7	17.2	0.7	26.7
	Counterfactual health literacy	18.4	11.6	0.7	12.0	0.7	16.5
	Counterfactual smoking behaviour	18.5	10.9	0.8	9.6	0.8	3.7
	Observed	20.8	-	0.8	-	0.9	-
Males							
	Counterfactual joint effect	10.6	39.0	0.5	38.1	0.5	48.0
	Counterfactual quality of social contacts	14.1	18.6	0.7	19.4	0.7	30.4
	Counterfactual health literacy	15.3	11.6	0.7	19.6	0.7	28.2
	Counterfactual smoking behaviour	15.4	10.9	0.7	16.6	1.0	3.8
	Observed	17.3	-	0.8	-	1.0	-

*Simulation: The transition rates for the observed data simulation are based on the values estimated in the Lifelines data. The transition rates for the counterfactual simulations assume the distribution of the modifiable factors for individuals with low education to be the same as for individuals with high education. The joint effect represents the situation in which all of the modifiable risk factors were changed.

[a] The absolute value of the difference between individuals with low and high education

[b] Percentage change from the observed simulation. Calculated by subtracting the difference between individuals with low and high education in the observed simulation from the difference seen in the counterfactual simulation and dividing by the difference in the observed simulation. Due to rounding, the percentages may differ from the value calculated using the differences presented in the table.

Table S12. Educational differences in the development of MDD over the life course stratified by sex. Sensitivity analysis using transition rates derived from a sample with a history of depression. Model parameters estimated using data from adults in the Lifelines Cohort Study collected between 2007 and 2017.

Sex	Simulation*	Difference [a] in life course prevalence (% points)	Percentage [b] change in counterfactual	Difference [a] in mean age of onset (years)	Percentage [b] change in counterfactual	Difference [a] in mean duration (years)	Percentage [b] change in counterfactual
Females							
	Counterfactual joint effect	13.0	37.7	0.4	48.7	1.4	36.4
	Counterfactual quality of social contacts	17.0	18.2	0.7	17.2	1.7	21.8
	Counterfactual health literacy	18.4	11.6	0.7	12.0	1.8	15.7
	Counterfactual smoking behaviour	18.5	10.9	0.8	9.6	2.1	6.0
	Observed	20.8	-	0.8	-	2.2	-
Males							
	Counterfactual joint effect	10.6	39.0	0.5	38.1	1.6	35.0
	Counterfactual quality of social contacts	14.1	18.6	0.7	19.4	1.9	19.1
	Counterfactual health literacy	15.3	11.6	0.7	19.6	2.0	15.4
	Counterfactual smoking behaviour	15.4	10.9	0.7	16.6	2.3	4.2
	Observed	17.291	-	0.8	-	2.4	-

*Simulation: The transition rates for the observed data simulation are based on the values estimated in the Lifelines data. The transition rates for the counterfactual simulations assume the distribution of the modifiable factors for individuals with low education to be the same as for individuals with high education. The joint effect represents the situation in which all of the modifiable risk factors were changed.

[a] The absolute value of the difference between individuals with low and high education

[b] Percentage change from the observed simulation. Calculated by subtracting the difference between individuals with low and high education in the observed simulation from the difference seen in the counterfactual simulation and dividing by the difference in the observed simulation. Due to rounding, the percentages may differ from the value calculated using the differences presented in the table.

Table S13. Descriptive characteristics of the study population used in the sensitivity analyses with and without MDD at baseline (n = 7,336).

**Characteristics**	**Lifelines population (n=15,199) [a]**	**Excluded population (n=7,863) [a]**	**Study population (n = 7,336) [a]**	**Population without MDD at baseline (n = 6,664) [a]**	**Population with MDD at baseline (n = 672) [a]**
Age (years), mean (SD)	44.7 (11.6)	44.9 (12.9)	44.3 (10.1)	44.4 (10.1)	43.4 (9.9)
Missing	0	0	0	0	0
Sex (female)	71.8	70.7	73.0	73.1	72.3
Missing	0	0	0	0	0
Education (years of education)					
Primary school not finished (5)	0.8	1.3	0.4	0.3	1.2
Primary education (6)	2.8	4.0	1.6	1.4	4.2
Lower or preparatory secondary vocational education (9)	13.6	15.3	11.8	11.1	18.8
Junior general secondary education (10)	15.9	16.8	14.9	14.5	19.1
Secondary vocational education or senior general secondary education (12)	40.2	38.6	42.0	42.3	38.8
Higher vocational education (15)	19.5	17.1	22.1	23.1	12.4
University education (16)	4.7	4.4	5.1	5.3	2.7
Missing	2.4	2.7	2.1	2.0	3.0
**Modifiable factors**					
Smoking					
Never smoker	33.5	31.3	35.9	35.8	36.9
Past smoker	30.2	28.1	32.4	33.1	25.6
Current smoker	28.9	30.0	27.7	27.0	35.0
Missing	7.4	10.6	3.9	4.1	2.5
Health literacy at T3 [b]					
Low	18.3	14.6	22.2	21.3	31.9
High	40.6	28.1	54.0	55.5	38.7
Missing	41.1	57.2	23.8	23.2	29.5

Quality of social contacts [c]					
Low	43.8	41.9	45.8	43.4	70.2
Middle	28.9	26.1	32.0	33.6	16.1
High	18.7	17.9	19.6	20.6	10.4
Missing	8.5	14.1	2.5	2.4	3.3

MDD: major depressive disorder; SD: standard deviation; [a] % are presented, unless indicated otherwise; [b] Measured with the Brief Health Literacy Screening (BHLS) on a continuous scale (3-15) which was categorized in low health literacy 3-12 and high health literacy 13-15; [c] Measured with the Social Production Function Instrument (SPF-IL) on a continuous scale (0–27) which was categorized as ‘low’(< =14 points),‘middle’(15–17 points) and ‘high’(18–27 points).

4. Supplementary Figures

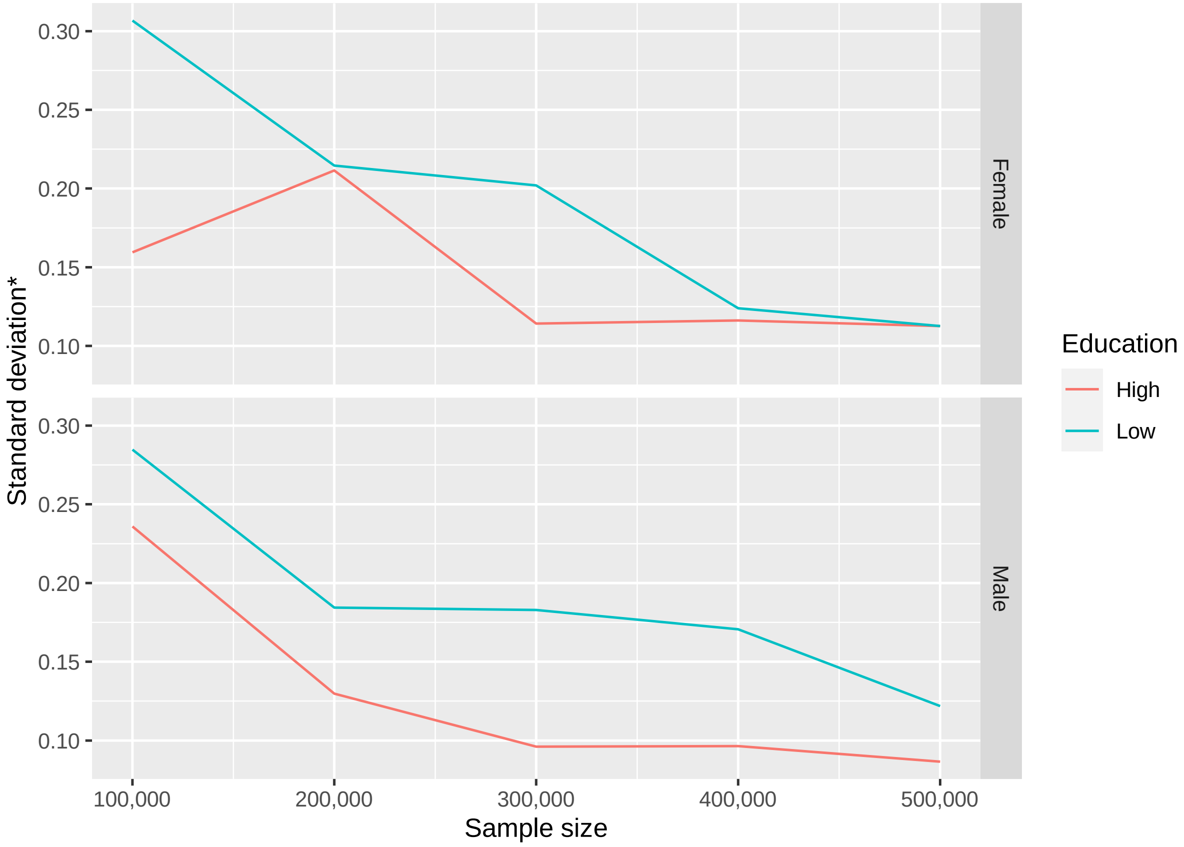

Fig S1. Monte Carlo variance associated with the estimates of the life course prevalence of MDD.
*****The simulations were repeated 20 times per sample size, and the standard deviation of the parameter was then estimated per sex- and education-groups.

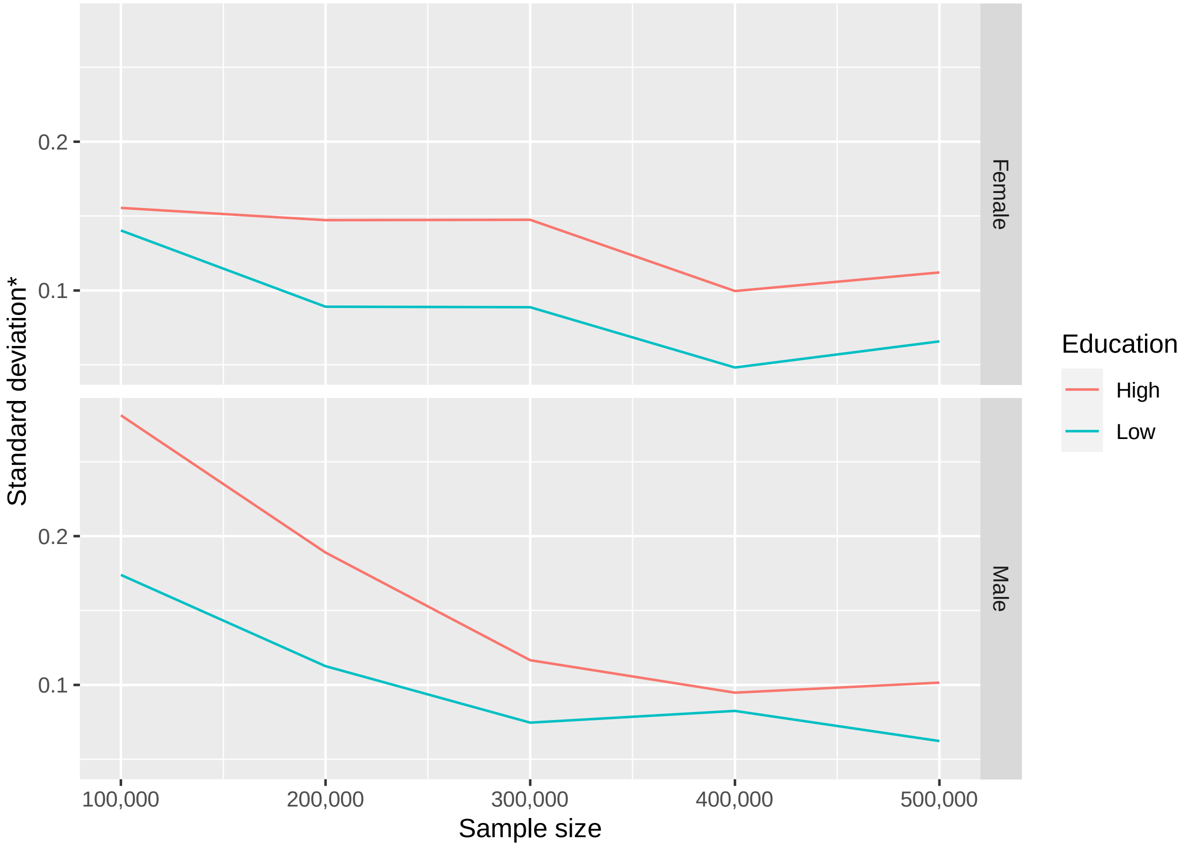

Fig S2. Monte Carlo variance associated with the estimates of the mean age of onset of MDD.
*****The simulations were repeated 20 times per sample size, and the standard deviation of the parameter was then estimated per sex- and education-groups.

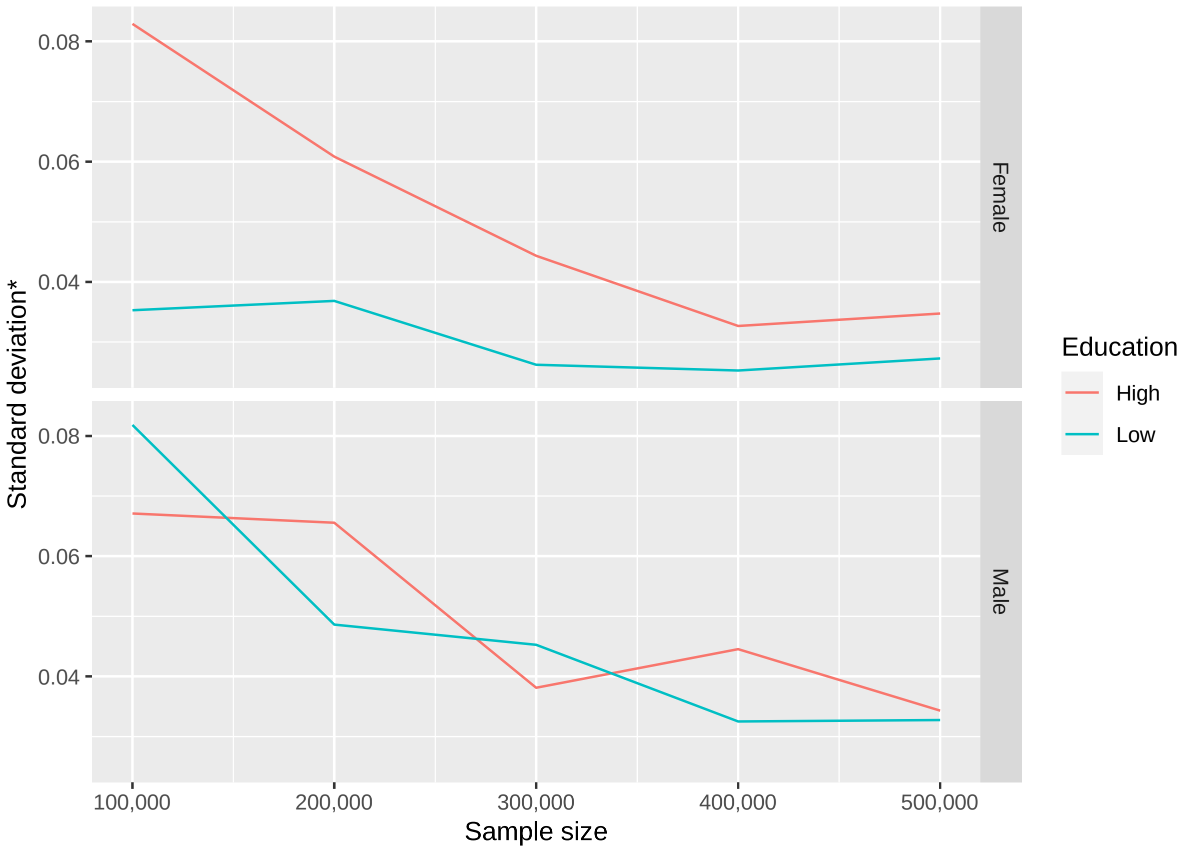

Fig S3. Monte Carlo variance associated with the estimates of the mean duration of MDD.
*The simulations were repeated 20 times per sample size, and the standard deviation of the parameter was then estimated per sex- and education-groups.

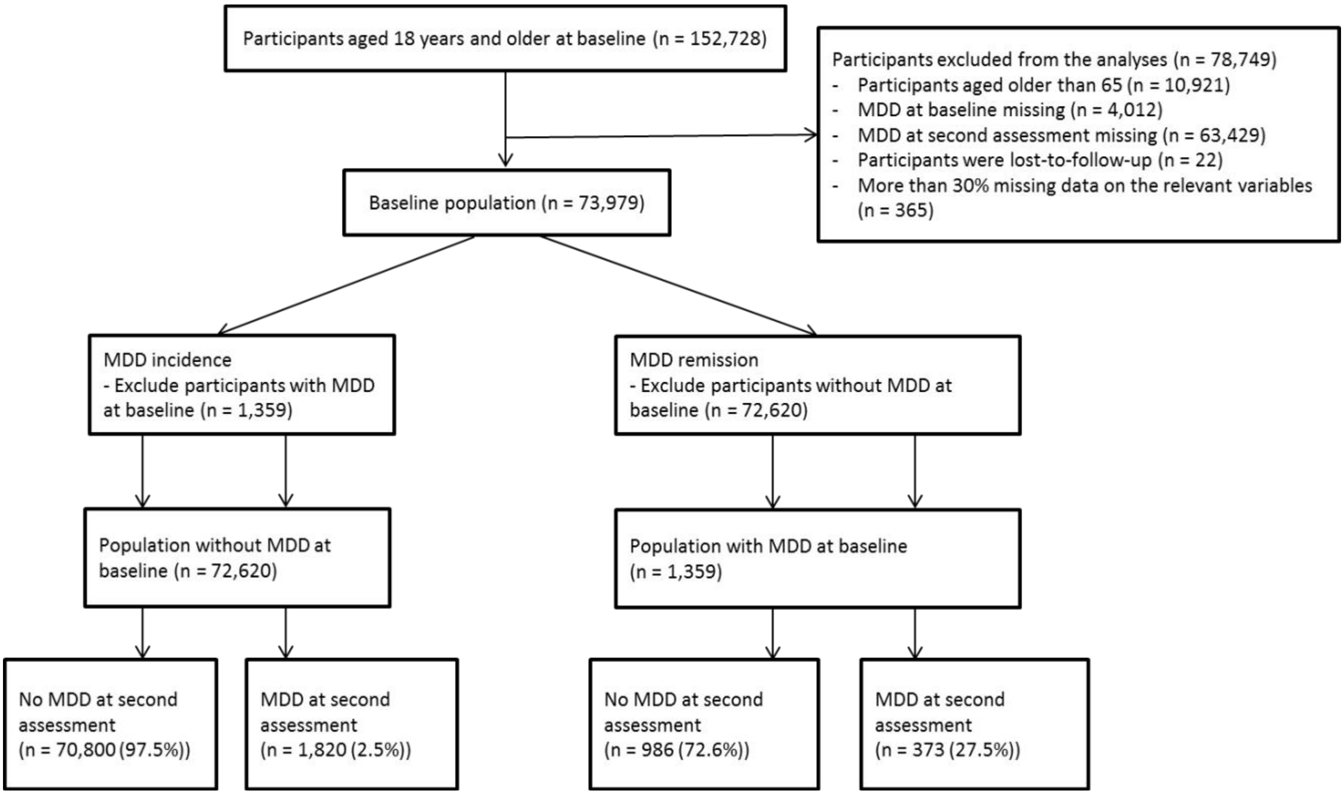

Fig S4. Flowchart of the population with and without MDD at baseline.

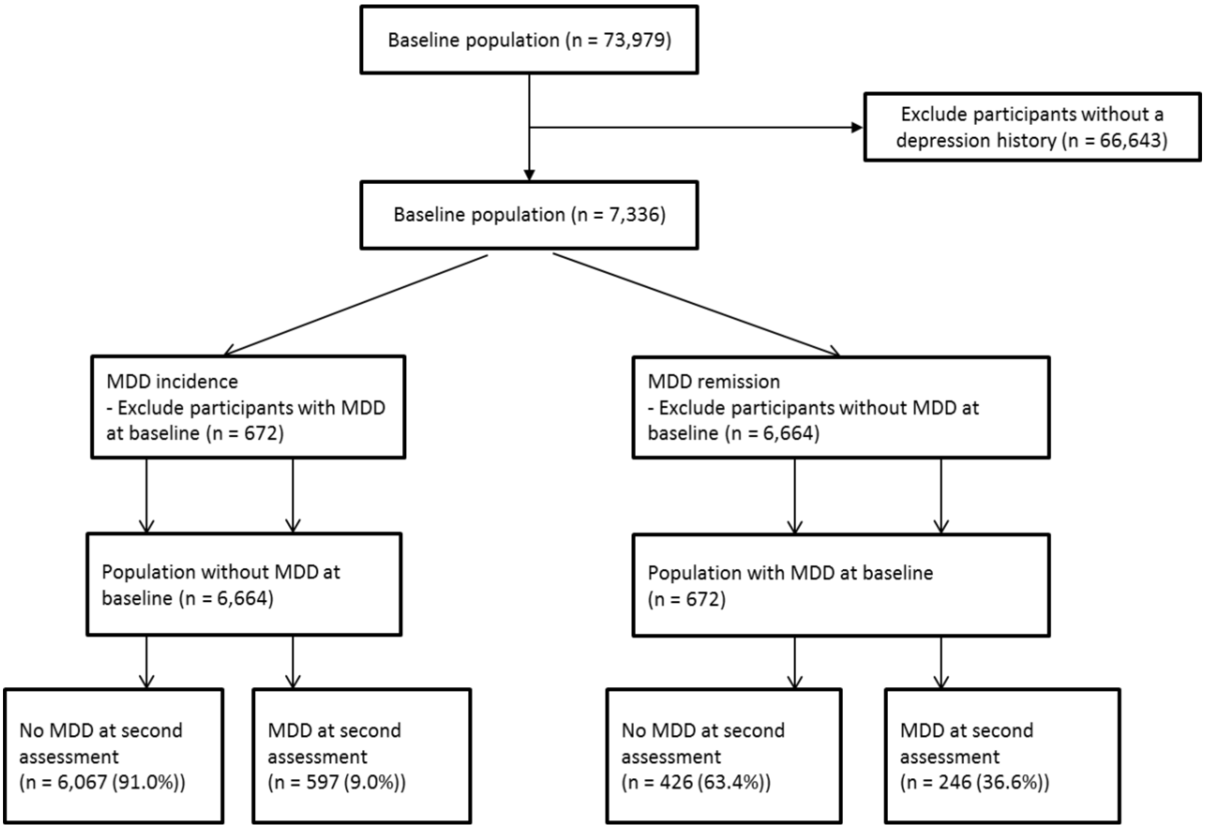

Fig S5. Flowchart of the population used for the sensitivity analysis.

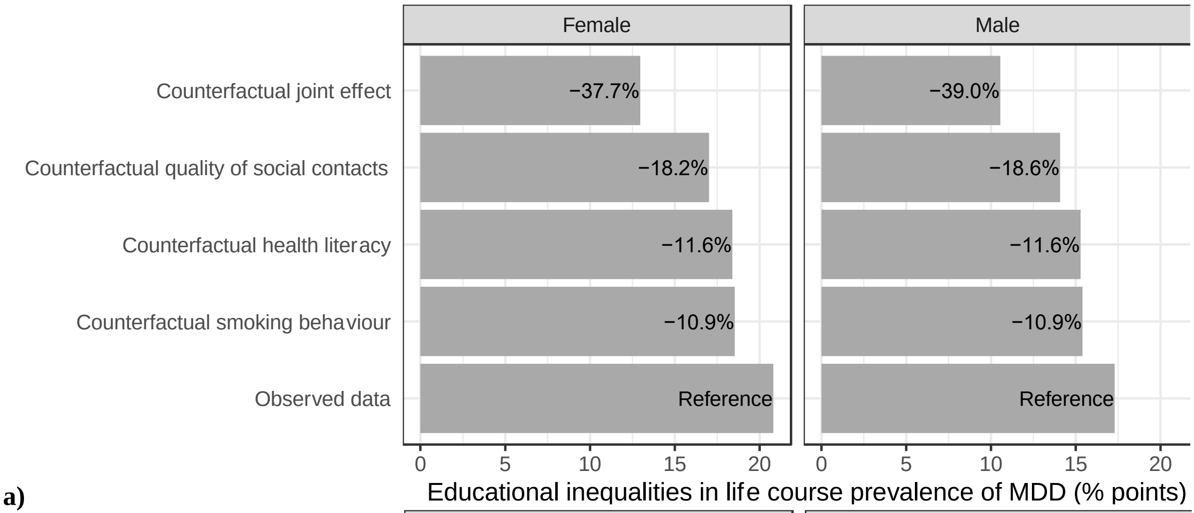

a)

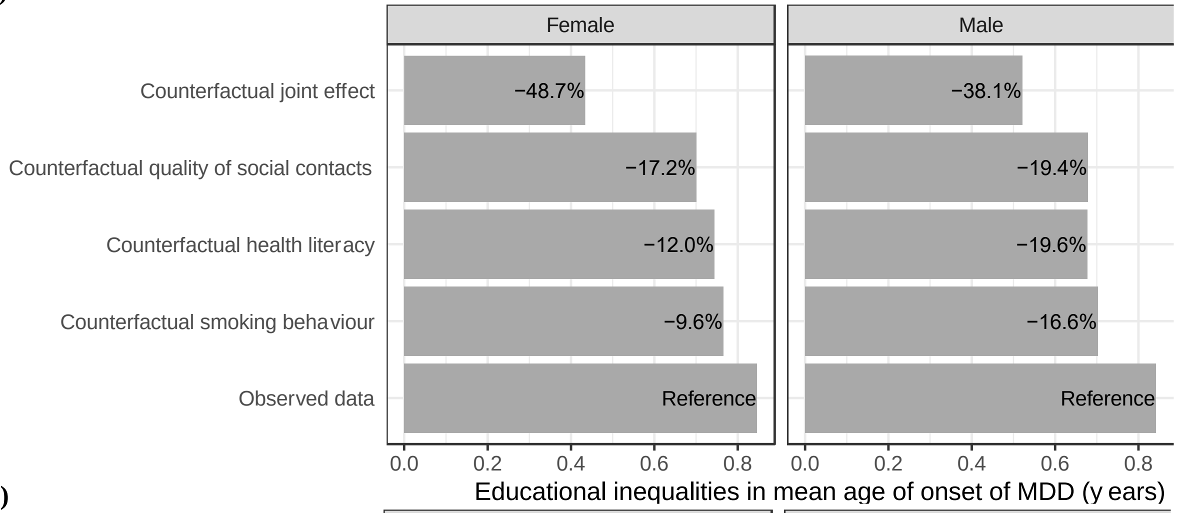

b)

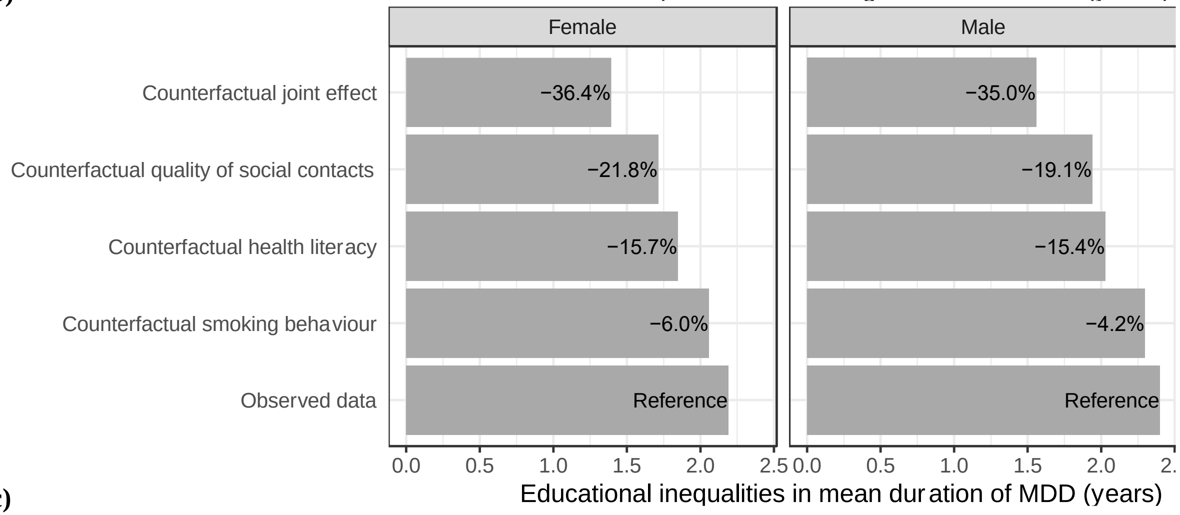

c)

Fig S6. Potential impact of modifiable factors on educational inequalities in a) life course prevalence, b) mean age of onset of, and c) mean duration of MDD between ages 18 and 65. Sensitivity analysis using transition rates derived from a sample with a history of depression. Model parameters estimated using data from adults in the Lifelines Cohort Study collected between 2007 and 2017.

*Note: The bars show a) the additional proportion of individuals with low education who ever experience MDD compared to individuals with high education, b) the difference in the mean age of onset between individuals with low and high education, and c) the additional number of years spent with MDD for individuals with low education compared to individuals with high education under different counterfactual conditions. Larger values for the difference in mean age of onset indicate earlier age of onset amongst individuals with low education. The percentages shown on the bars represent the percentage reduction under the given counterfactual.

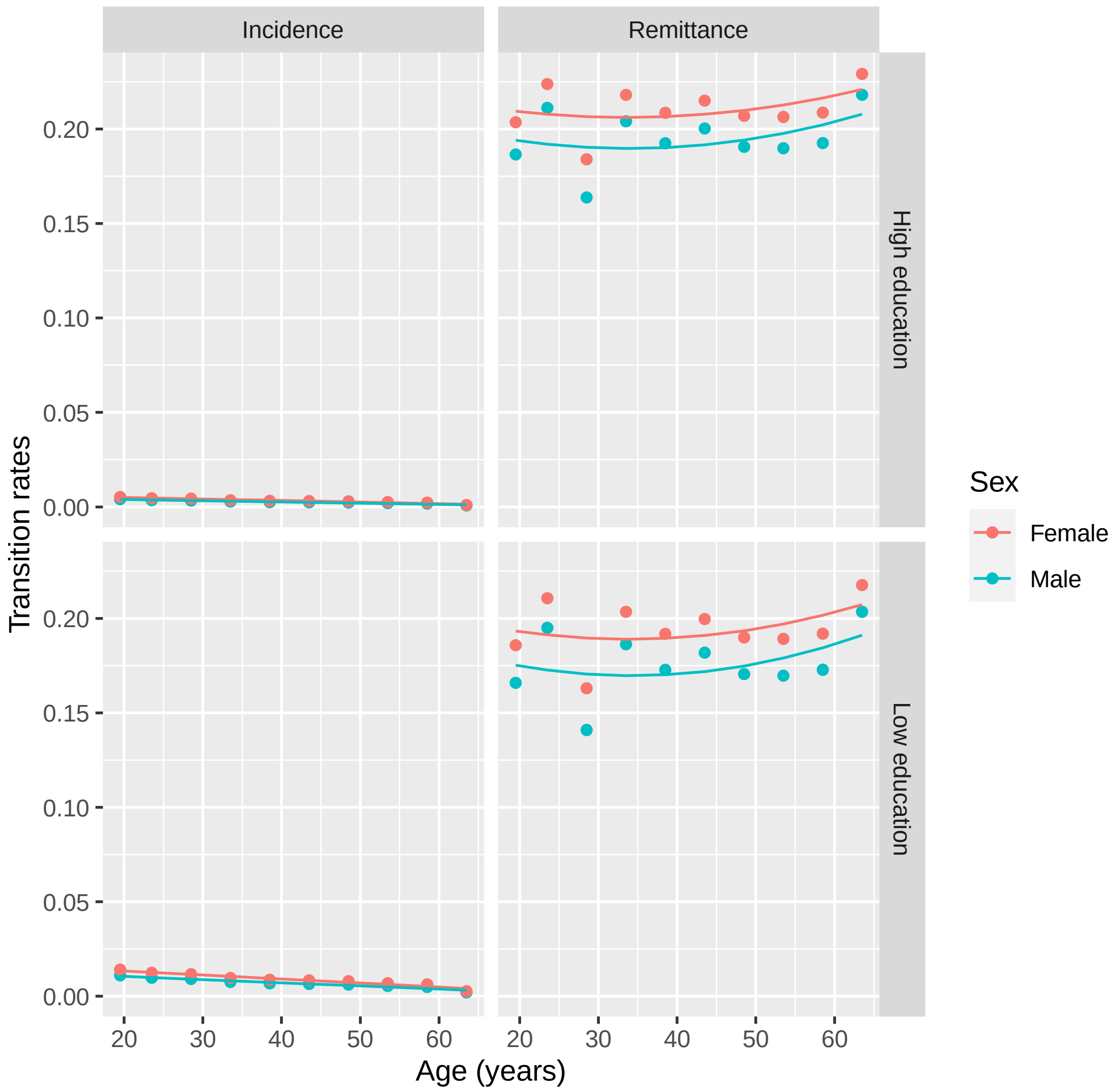

Fig S7. Observed (points) and estimated (lines) values of transition rates for males and females by education level. Model parameters estimated using data from adults in the Lifelines Cohort Study collected between 2007 and 2017.
